# Toxic Effects of the Major Components of Diesel Exhaust in Human Alveolar Basal Epithelial Cells (A549)

**DOI:** 10.3390/ijms17091393

**Published:** 2016-08-26

**Authors:** Pavel Rossner, Simona Strapacova, Jitka Stolcpartova, Jana Schmuczerova, Alena Milcova, Jiri Neca, Veronika Vlkova, Tana Brzicova, Miroslav Machala, Jan Topinka

**Affiliations:** 1 Institute of Experimental Medicine, Academy of Sciences of the Czech Republic, Videnska 1083, 142 20 Prague, Czech Republic; jitka.stolcpartova@biomed.cas.cz (J.St.); schmucze@yahoo.com (J.Sc.); milcova@biomed.cas.cz (A.M.); veronika.vlkova@biomed.cas.cz (V.V.); tana.brzicova@biomed.cas.cz (T.B.); jtopinka@biomed.cas.cz (J.T.); 2 Veterinary Research Institute, Hudcova 70, 621 00 Brno, Czech Republic; strapacova@vri.cz (S.S.); neca@vri.cz (J.N.); machala@vri.cz (M.M.); 3 Institute for Environmental Studies, Faculty of Science, Charles University, Benatska 2, 128 01 Prague 2, Czech Republic

**Keywords:** benzo[a]pyrene, 1-nitropyrene, 3-nitrobenzanthrone, bulky DNA adducts, oxidative damage, gene expression

## Abstract

We investigated the toxicity of benzo[a]pyrene (B[a]P), 1-nitropyrene (1-NP) and 3-nitrobenzanthrone (3-NBA) in A549 cells. Cells were treated for 4 h and 24 h with: B[a]P (0.1 and 1 μM), 1-NP (1 and 10 μM) and 3-NBA (0.5 and 5 μM). Bulky DNA adducts, lipid peroxidation, DNA and protein oxidation and mRNA expression of *CYP1A1*, *CYP1B1*, *NQO1*, *POR*, *AKR1C2* and *COX2* were analyzed. Bulky DNA adducts were induced after both treatment periods; the effect of 1-NP was weak. 3-NBA induced high levels of bulky DNA adducts even after 4-h treatment, suggesting rapid metabolic activation. Oxidative DNA damage was not affected. 1-NP caused protein oxidation and weak induction of lipid peroxidation after 4-h incubation. 3-NBA induced lipid peroxidation after 24-h treatment. Unlike B[a]P, induction of the aryl hydrocarbon receptor, measured as mRNA expression levels of *CYP1A1* and *CYP1B1*, was low after treatment with polycyclic aromatic hydrocarbon (PAH) nitro-derivatives. All test compounds induced mRNA expression of *NQO1*, *POR*, and *AKR1C2* after 24-h treatment. *AKR1C2* expression indicates involvement of processes associated with reactive oxygen species generation. This was supported further by *COX2* expression induced by 24-h treatment with 1-NP. In summary, 3-NBA was the most potent genotoxicant, whereas 1-NP exhibited the strongest oxidative properties.

## 1. Introduction

Environmental air pollution affects most of the human population. In industrialized countries, air pollutants originate from three major sources: domestic heating, industrial activities, and traffic. Despite substantial technological advances resulting in decreased fuel consumption and lower emissions, road traffic remains a significant source of air pollution, particularly in metropolitan areas. Recently, the International Agency for Research on Cancer (IARC) classified outdoor air pollution as “carcinogenic to humans” (Group 1) [1,2].

From a complex mixture of chemicals and particles produced by incomplete combustion of organic material, including oil and oil products, polycyclic aromatic hydrocarbons (PAHs) are noteworthy due to their metabolism to reactive compounds that can bind to nucleic acids and proteins, cause their damage and/or loss of function, and induce mutations. Apart from binding to macromolecules resulting in formation of PAH–DNA adducts or protein adducts, some PAHs may generate reactive oxygen species (ROS) and thus induce oxidative stress. Nitro-PAHs may be produced by direct sources (diesel and gasoline exhaust), but may also be generated by reactions of PAHs with oxides of nitrogen in the atmosphere [3]. Nitro-PAHs are characterized by persistence in the environment and high mutagenic and carcinogenic activities in model systems. Their concentrations in the environment are lower than those of parent PAHs, but some nitro-PAHs have been shown to act as direct mutagens that do not require metabolic activation [3].

Benzo[a]pyrene has been classified as carcinogenic to humans (IARC, Group 4) [4]. Three pathways have been proposed for metabolic activation of B[a]P: (i) cytochrome P450-catalyzed conversion of B[a]P to epoxide that is further metabolized by epoxide hydrolase and CYP1A1 to diol epoxide, which binds to DNA; (ii) P450 peroxidase-catalyzed activation to reactive radical cations that bind to DNA; (iii) dihydrodiol dehydrogenase-catalyzed conversion of dihydrodiols to catechol. The latter is unstable and undergoes auto-oxidation first to a semiquinone radical and hydrogen peroxide followed by formation of *o*-quinone and the superoxide anion. Eventually, these events lead to redox-cycling and induction of oxidative stress [5].

1-Nitropyrene (1-NP) is the most abundant nitro-PAH in emissions from diesel engines. It is considered to be the main contributor of direct-acting mutagenicity of diesel exhaust as assessed by the Ames test [6]. 1-NP is metabolized by three major pathways. The first pathway utilizes cytochrome P450-mediated ring oxidation to non-K-region phenols and K-region oxides; K-region oxides are converted further to trans-dihydrodiols or phenols that bind to macromolecules. The second pathway involves nitroreduction in one- or two-electron steps to form 1-nitrosopyrene and *N*-hydroxy-1-aminopyrene or 1-aminopyrene. The third pathway is a combination of ring oxidation and nitroreduction followed by acetylation [7,8]. 1-NP also induces ROS production by induction of endoplasmic reticulum (ER) stress mediated by 1-NP metabolites formed during nitroreduction [9]. Another source of ROS is a reactive intermediate of 1-NP, the *N*-hydroxy radical, which undergoes auto-oxidation coupled with generation of the superoxide radical and hydrogen peroxide [10]. 1-NP has been classified as “probably carcinogenic to humans” (IARC, Group 2A, [11]).

3-Nitrobenzanthrone (3-NBA) is one of the most potent mutagens and a potential human carcinogen (IARC Group 2B, possibly carcinogenic to humans, [2]). Apart from incomplete combustion of fossil fuels, 3-NBA can also originate from the reaction of benzanthrone (a pollutant abundant in exhaust gas and particles from diesel engines) with nitrogen oxides in the atmosphere [11]. In organisms, 3-NBA is metabolized by nitroreduction catalyzed by xanthine oxidase (XO), NAD(P)H:quinone oxidoreductase (NQO1) and NAPDH:cytochrome P450 oxidoreductase (POR) to *N*-hydroxy-3-aminobenzanthrone (N-OH-ABA). The latter can be metabolized further by two pathways: the major pathway results in formation of non-acetylated 3-NBA DNA adducts, whereas the minor pathway leads to acetylated 3-NBA DNA adducts [12,13]. Furthermore, N-OH-ABA can induce production of the superoxide radical and hydrogen peroxide as well as subsequent oxidative damage to macromolecules [13]. Unlike B[a]P and 1-NP, CYP enzymes do not contribute significantly to 3-NBA activation [14].

Inhalation is a primary route of exposure of humans to PAHs and their derivatives. Hence, studies on cell models derived from lung tissue could provide the most relevant information on the negative effects of these compounds to humans. A549 is a human lung adenocarcinoma cell line commonly used as a model in genetic toxicology. Being a cancer cell line, this model is not suitable for cytogenetic applications, or studies of cell cycle regulation. However, its morphology and many basic cellular functions are comparable with normal alveolar epithelial type-II cells. Hence, these cells are useful for studies of metabolic processes associated with exposure to xenobiotics and their toxic effects [15,16].

Here, we aimed to compare toxic effects (including damage to DNA, lipids and proteins as well as mRNA expression of selected relevant genes) in A549 cells treated with model PAH (B[a]P) and nitro-PAHs (1-NP, 3-NBA). The genes included those encoding enzymes participating in the metabolism of xenobiotics (CYP1A1, CYP1B1, NQO1, POR) and/or associated with oxidative stress (aldo-keto reductase (AKR1C2), cyclooxygenase-2 (COX2)). We tested two concentrations of each compound. To account for the different kinetics of biologic effects induced by test compounds, we treated cells for two different time intervals (4 h and 24 h).

## 2. Results

### 2.1. Bulky DNA Adducts

**Figure 1:**
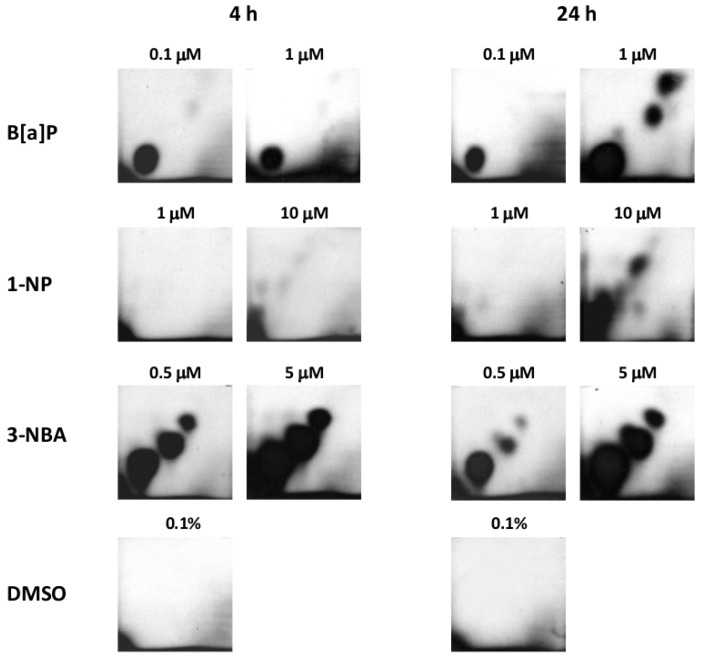
Autoradiographs of TLC maps of ^32^P-labeled DNA digests after incubation of A549 cells with various concentrations of benzo[a]pyrene, 1-nitropyrene and 3-nitrobenzanthrone for 4 h and 24 h. Control panels depict analyses of A549 cells treated withdimethylsulfoxide. DNA (5 µg) was analyzed using the nuclease P1 method of sensitivity enhancement. Screen-enhanced autoradiography was performed at −80 °C for 24 h.

**Figure 2:**
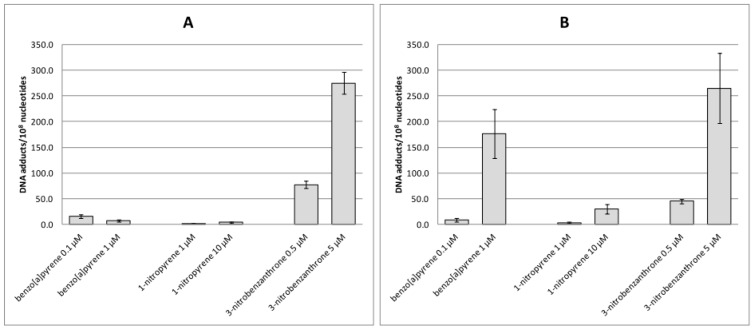
Levels of bulky DNA adducts/10^8^ nucleotides induced after treatment of A549 cells with benzo[a]pyrene, 1-nitropyrene and 3-nitrobenzanthrone for 4 h (**A**) and 24 h (**B**). Data represent mean values ± standard deviation from two triplicates from two independent experiments (analyzed as *n* = 6) (negative control subtracted). Based on the comparison with autoradiographs (Figure 1), bulky DNA adduct levels induced by 1-nitropyrene were negligible.

Four-hour treatment of A549 cells with 0.1 and 1 µM of B[a]P induced one major adduct spot whereas, after 24-h treatment with 1 µM of B[a]P, three additional minor spots representing three other adduct types were detected (Figure 1). With the exception of the 10-µM concentration after 24-h incubation (when multiple DNA adduct spots were visible on autoradiographs), 1-NP induced weak adduct spots. In contrast, 0.5 µM and 5 µM 3-NBA induced 3–4 strong adduct spots after 4-h and 24-h treatment (Figure 1). Levels of bulky DNA adducts after treatment with 3-NBA (5 μM) exceeded 275 adducts/10^8^ nucleotides (Figure 2A). After 24-h treatment with 1 μM B[a]P, levels of DNA adducts reached 175 adducts/10^8^ nucleotides, whereas the 0.1 μM concentration of B[a]P induced much lower levels of DNA adducts (≈10 adducts/10^8^ nucleotides). Interestingly, although levels of DNA adducts induced by 3-NBA after 24-h treatment were high, we observed almost 50% reduction in their levels after incubation with 3-NBA (0.5 μM). Levels of DNA adducts induced by 1-NP were low even after 24-h incubation with the compound, when the higher test dose (10 μM) induced almost 30 adducts/10^8^ nucleotides (Figure 2B).

### 2.2. Oxidative Damage to DNA, Lipids and Proteins

**Figure 3:**
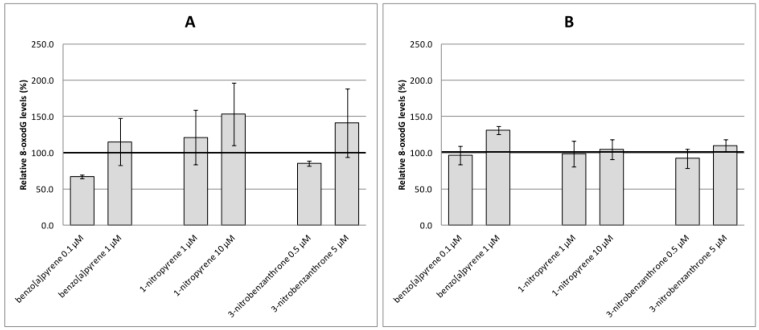
8-oxodG levels relative to the control (%) induced after treatment of A549 cells with benzo[a]pyrene, 1-nitropyrene and 3-nitrobenzanthrone for 4 h (**A**) and 24 h (**B**). Data represent mean values ± standard deviation from two triplicates from two independent experiments (analyzed as *n* = 6). The control level of 8-oxodG is represented by an emboldened horizontal line.

**Figure 4:**
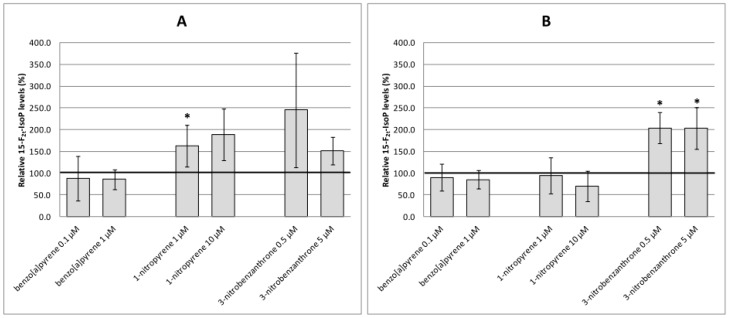
15-F2t-IsoP levels relative to the control (%) induced after treatment of A549 cells with benzo[a]pyrene, 1-nitropyrene and 3-nitrobenzanthrone for 4 h (**A**) and 24 h (**B**). Data represent mean ± standard deviation from two triplicates from two independent experiments (analyzed as *n* = 6). Asterisks denote significant (* *p* < 0.05) changes. The control level of 15-F2t-IsoP is represented by an emboldened horizontal line. 1-nitropyrene (1 µM, 4 h) and 3-nitrobenzanthrone (0.5 µM and 5 µM, 24 h) significantly increased 15-F2t-IsoP levels.

**Figure 5:**
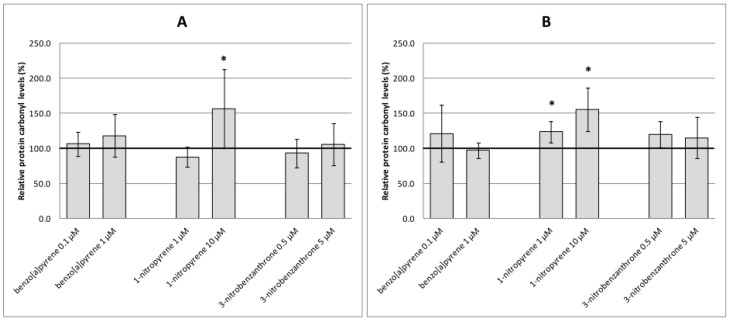
Levels of protein carbonyls relative to the control (%) induced after treatment of A549 cells with benzo[a]pyrene, 1-nitropyrene and 3-nitrobenzanthrone for 4 h (**A**) and 24 h (**B**). Data represent mean ± standard deviation from two triplicates from two independent experiments (analyzed as *n* = 6). Asterisks denote significant (* *p* < 0.05) changes. The control level of protein carbonyls is represented by an emboldened horizontal line. 1-nitropyrene (10 µM, 4 h; 1 µM and 10 µM, 24 h) significantly increased protein carbonyl levels.

Analyses of oxidative damage (Figure 3, Figure 4 and Figure 5) indicate relatively weak and non-consistent effects of test compounds on markers of oxidative damage. None of the time intervals and test (non-cytotoxic) doses of B[a]P increased levels of oxidative damage to DNA (8-oxodG, Figure 3A,B), lipids (15-F_2t_-IsoP, Figure 4A,B) or proteins (protein carbonyls, Figure 5A,B) significantly. Most of the significant biological effects could be ascribed to 1-NP treatment. This compound increased levels of lipid peroxidation after 4-h treatment (Figure 4A) and induced protein oxidation after 4-h and 24-h incubation (Figure 5A,B). However, no significant effect on DNA oxidation (Figure 3A,B) or lipid peroxidation after 24-h treatment (Figure 4B) was detected. 3-NBA significantly induced lipid peroxidation after 24-h treatment; levels of 15-F_2t_-IsoP increased to 200% of the relative control level, and were comparable for both test concentrations (Figure 4B). Levels of lipid peroxidation after 4-h treatment with 3-NBA were elevated, but the increase was not significant (Figure 4A). We did not detect an effect of this compound on oxidation of DNA or protein (Figure 3A,B and Figure 5A,B).

### 2.3. mRNA Expression of Selected Genes

**Figure 6:**
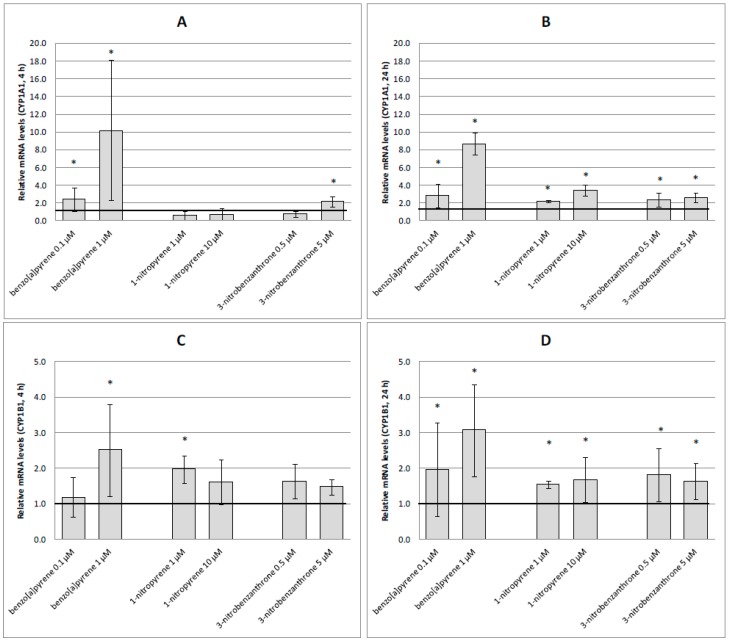

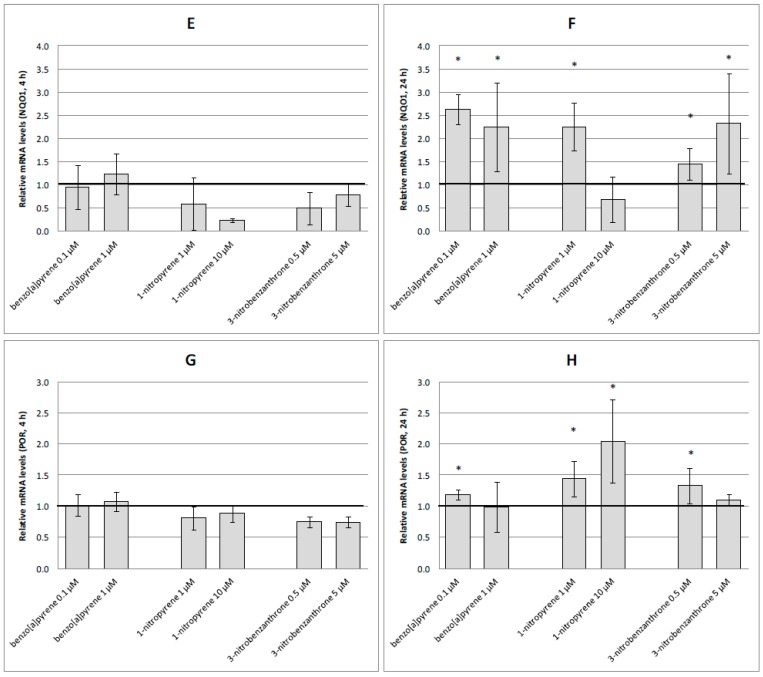
Relative mRNA levels (fold changes relative to the control) induced after treatment of A549 cells with benzo[a]pyrene, 1-nitropyrene and 3-nitrobenzanthrone. (**A**) *CYP1A1*, 4 h; (**B**) *CYP1A1*, 24 h; (**C**) *CYP1B1*, 4 h; (**D**) *CYP1B1*, 24 h; (**E**) *NQO1*, 4 h; (**F**) *NQO1*, 24 h; (**G**) *POR*, 4 h; (**H**) POR, 24 h. Data represent mean values ± standard deviation from two triplicates from two independent experiments (analyzed as *n* = 6). Asterisks denote significant (* *p* < 0.05) gene expression changes relative to the control. Control expression level of the respective gene is represented by an emboldened horizontal line.

Expression levels of genes encoding metabolic activation enzymes are shown in Figure 6. As expected, B[a]P induced *CYP1A1* expression in both tested doses and intervals (Figure 6A,B). Similarly, this compound induced *CYP1B1* expression, although no significant effect was observed for the 4-h treatment with 0.1 μM B[a]P. Both tested nitro-PAHs required the 24-h treatment to exert consistent induction of *CYP1A1* and *CYP1B1* expression (Figure 6B,D), although 3-NBA (5 μM) induced *CYP1A1* expression (Figure 6A) and 1-NP (1 μM) induced *CYP1B1* expression (Figure 6C) after the 4-h treatment. However, the induction levels were weak compared to those after the B[a]P treatment. None of the tested compounds had significant effect on *NQO1* and *POR* expression after the 4-h treatment (Figure 6E,G). In most cases, the longer treatment period increased expression of these mRNAs significantly (Figure 6F,H). For *NQO1*, no effects were observed for 1-NP at 10 μM (Figure 6F). Substantial differences in induction levels between compounds were not observed, but B[a]P tended to induce higher expression of *NQO1*. Highest levels of *POR* expression were induced after 1-NP treatment; for B[a]P and 3-NBA, only the lower test dose induced significant changes in gene expression (Figure 6H).

**Figure 7:**
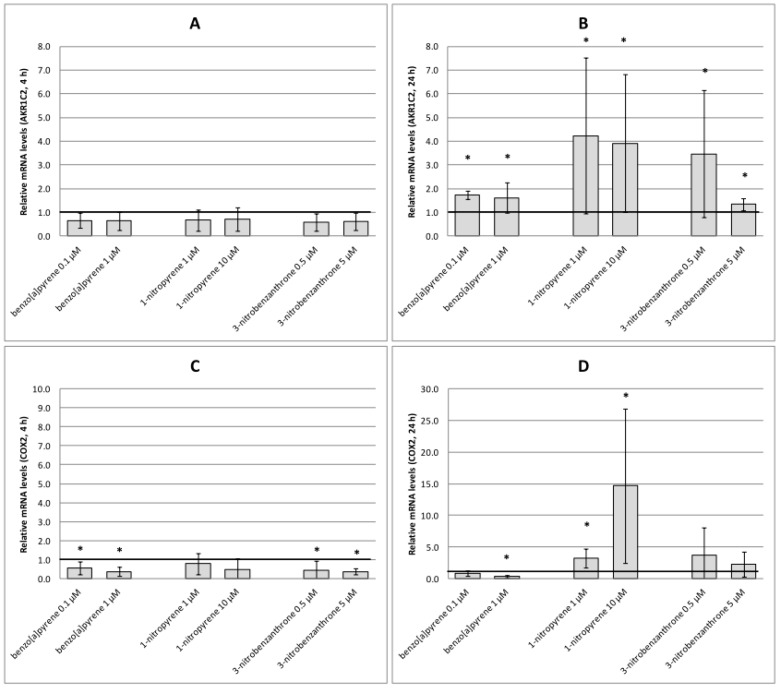
Relative mRNA levels (fold changes relative to the control) induced after treatment of A549 cells with benzo[a]pyrene, 1-nitropyrene and 3-nitrobenzanthrone. (**A**) *AKR1C2*, 4 h; (**B**) *AKR1C2*, 24 h; (**C**) *COX2*, 4 h; (**D**) *COX2*, 24 h. Data represent mean values ± standard deviation from two triplicates from two independent experiments (analyzed as *n* = 6). Asterisks denote significant (* *p* < 0.05) gene expression changes relative to the control. The control expression level of the respective gene is represented by an emboldened horizontal line.

Although none of the test compounds increased *AKR1C2* expression after 4-h treatment (Figure 7A), induction was observed for all chemicals and doses after the longer treatment period. 1-NP was identified as the most potent inducer of *AKR1C2* expression (Figure 7B). Four-hour treatment of A549 cells with B[a]P and 3-NBA resulted in a significant decrease in *COX2* expression (Figure 7C). This decrease was also observed after 24-h treatment with 1 μM B[a]P. 1-NP, particularly at the higher test dose, was a strong inducer of *COX2* after 24-h incubation (Figure 7D).

## 3. Discussion

In the present study, we aimed to characterize differences in the response of A549 cells treated with a model PAH (B[a]P) and nitro-PAHs (1-NP, 3-NBA), i.e., compounds with different metabolism. We selected bulky DNA adducts and markers of oxidative damage as endpoints for the comparison of toxic effects of test compounds. Furthermore, we analyzed mRNA expression of selected genes to identify potential mechanisms responsible for changes in levels of studied endpoints. The rationale behind the selection of compounds was the fact that B[a]P was the only PAH classified by IARC as being carcinogenic to humans (Group 1) and nitro-PAHs were pollutants associated mostly with diesel exhaust and thus with traffic.

Selection of a suitable cell model is critical for interpretation of the results of in vitro toxicity testing. Lungs are the primary target for air pollutants, so cells of lung-tissue origin are a logical choice for investigation of the toxic effects of PAHs and nitro-PAHs. Results are affected mainly by the activity of metabolic activation enzymes in a selected cell line. A recent study compared genotoxic responses to B[a]P in three human cell lines (including A549 cells) and observed extreme variability for various markers (including DNA adducts) [17]. Authors observed a moderate increase in levels of BPDE–*N*^2^–dGuo adducts in A549 cells after 24-h treatment, but only for low doses of the compound (0.2 μM); at higher B[a]P concentrations, levels of DNA adducts decreased. While is not in agreement with our present data, it should be noted that in our study not only specific BPDE-*N*^2^-dGuo, but bulky DNA adducts, were analyzed. Furthermore, previously we analyzed levels of bulky DNA adducts after treatment of A549 cells with B[a]P concentrations ranging from 1 nM to 10 μM, and detected a decrease in levels of DNA adducts but only at >1 μM [18]. The decrease probably reflects general toxicity of high B[a]P doses negatively impacting cellular functions. On the other hand, after 4-h treatment, levels of bulky DNA adducts were low for both test concentrations of B[a]P. In general, levels of bulky DNA adducts are dependent upon the activity of CYP1A1, a key enzyme responsible for the metabolism of B[a]P [5]. Its expression is further modulated by the activity of transcription factor Nrf2 [19,20]. Although at the 4-h time-point we detected a significant increase in the expression of *CYP1A1* mRNA at a low B[a]P dose, the metabolic activity of cells (i.e., levels of the active CYP1A1 protein) was not probably sufficient to increase levels of bulky DNA adducts.

We observed very low levels of bulky DNA adducts induced by 1-NP, even after 24-h treatment with a higher dose of the compound. It has been demonstrated that 1-NP is activated by human CYP1 enzymes (especially CYP1B1) to exert genotoxicity in the *umu* test [21]. 1-NP induces only low DNA adduct formation and also low levels of *CYP1A1* and *CYP1B1* mRNA in human bronchial epithelial (BEAS-2B) cells [22]; another study showed that induction of expression of *CYP1A1* and *CYP1B1* in these cells was very weak [23]. This observation is in agreement with our data, which indicated low induction of *CYP1B1* mRNA even after 24-h treatment with 10 µM 1-NP. Interestingly, this treatment, which resulted in the highest levels of bulky DNA adducts induced by 1-NP, was also associated with almost fourfold induction of *CYP1A1* expression. Thus, the low DNA adduct levels induced by 1-NP in A549 cells could be caused by low expression of the gene encoding the CYP1B1 enzyme in this cell line. Our data also suggest that CYP1A1 may play a part in the formation of 1-NP-induced bulky DNA adducts.

3-NBA is a potent mutagen and has been shown repeatedly to induce DNA adducts in various model systems (reviewed in [11]). However, we identified only two studies analyzing this endpoint in A549 cells. Nagy et al. observed a linear dose–response relationship between 24-h exposure to 3-NBA (2, 5, 10 and 20 μM) and levels of DNA adducts [24]. In another study, 18-h exposure of A549 cells to 3-NBA (10 μM) also resulted in induction of DNA adducts [25]. It is worth mentioning that in our study, a 3-NBA concentration as low as 0.5 μM increased levels of DNA adducts. In addition, unlike other test compounds, the effect was observed after 4-h treatment. Theoretically, greater genotoxic potency, consistent with similar observations in BEAS-2B cells [22], may be associated with a different type of metabolic activation of 3-NBA, in which NQO1 and POR enzymes play an important part in conversion of 3-NBA to N-OH-ABA [12,14], whereas CYP enzymes are not considered to contribute significantly to 3-NBA activation [14]. 3-aminobenzanthrone, a product of the metabolic activation of 3-NBA, has been shown to be converted further by CYP1A1 to N-OH-ABA, thereby enhancing its genotoxicity [26,27]. The authors demonstrated that 3-NBA induces the protein expression and activity of CYP1A1 in rats [26]. However, our results did not show a significant increase in levels of *NQO1* and *POR* mRNA after 4-h treatment with 3-NBA; CYP1A1 expression showed a modest increase only after treatment with higher doses of the compound. Thus, in our experimental system, high levels of bulky DNA adducts induced by the 4-h treatment with 3-NBA are not likely to be associated with mRNA expression of *NQO1*, *POR* and *CYP1A1* genes. Twenty-four-hour treatment with 3-NBA resulted in a significant induction in levels of *NQO1*, *CYP1A1* and (for the lower dose of compound) *POR*, although the increase was modest and cannot probably explain strong induction of bulky DNA adducts. Further information might be obtained from analyses of protein expression because basal levels of these enzymes sufficient for metabolic activation of 3-NBA might be present in A549 cells.

Theoretically, all test compounds could induce ROS generation and, therefore, oxidative damage to macromolecules, though the mechanism of action differs. For B[a]P, this mechanism includes the activity of dihydrodiol dehydrogenases, including AKR1C2 [5]. Indeed, in our study, we observed a significant increase in expression of *AKR1C2* mRNA after 24-h treatment with both doses of B[a]P, although the increase is relatively modest (~1.8-fold) and its biological meaning might be limited. Moreover, similar effects were found after treatment with nitro-PAHs. These observations may be because regulation of *AKR1C2* expression is probably mediated by the antioxidant response of cells rather than by a xenobiotic response [5]. Thus, our results on *AKR1C2* expression indicate that some levels of ROS are generated after treatment of A549 cells with B[a]P and nitro-PAHs. These processes may be responsible for the induction of lipid peroxidation and oxidative damage to proteins after treatment with nitro-PAHs. Considering the fact that B[a]P had no effect on oxidative damage of either macromolecule, we may conclude that nitro-PAHs tested were stronger pro-oxidants under our experimental conditions. 1-NP clearly induces oxidative stress by ROS production [9,28], but the mechanisms by which ROS are produced are not conclusively identified. It has been reported that 1-nitrosopyrene, a metabolite of 1-NP, can induce oxidative damage [10]. The authors of another study [9] argued that increased ROS levels upon 1-NP treatment were associated with ER stress induced by metabolites of 1-NP during nitroreduction. 3-NBA may contribute to ROS generation due to the activity of enzymes participating in its metabolic activation. Xanthine oxidase reduces molecular oxygen to the superoxide radical and hydrogen peroxide, thus generating ROS [16]. In another study, a mechanism of ROS generation involving N-OH-ABA was proposed [13]. N-OH-ABA is formed from 3-NBA by the action of NQO1, POR or XO [11]; in the presence of NADH and Cu(II), N-OH-ABA may be auto-oxidized, resulting in superoxide generation and further dismutation of superoxide to hydrogen peroxide.

Despite the findings mentioned above, we did not detect oxidative DNA damage after treatment with any compound. The literature suggests that, in A549 cells, DNA is oxidized only after application of higher doses of the compound (i.e., 2 μM for 14 h; [17]). ROS production induced by B[a]P is also dependent on the cell type because, in our previous study on human embryonic lung fibroblasts, we did not detect 8-oxodG formation even after a 48-h application of 100 μM B[a]P [29]. However, Bolck et al. reported ROS formation in human immortalized keratinocytes even after very short treatment with 1 μM B[a]P [30]. 1-NP can also induce oxidative DNA damage as shown, for example, in human umbilical vein endothelial cells at doses <10 μM [9], or in naked DNA in the presence of NADPH [10]. In HaCaT keratinocytes, 1-NP induced oxidative DNA damage but only in the presence of UV-light [31]. In another study, exposure to 1-NP resulted in formation of 8-OH-dG in A549 cells but only after application of very high doses of the compound (above 250 μM). The authors argue that 1-NP causes ROS generation, but the repair mechanisms of cells can remove oxidized bases provided that 1-NP concentrations are not too high [28]. This observation may explain why, in our study, no increase in oxidative DNA damage was detected, whereas oxidative damage to lipids and proteins that are not actively repaired by cellular mechanisms was found. 3-NBA has also been shown to induce oxidative DNA damage as measured by the Comet assay in A549 cells after treatment with 10 µM of 3-NBA for 18 h [25]. In another study, 1-h incubation of cells with 0.1 and 6 µM 3-NBA did not significantly increase oxidative DNA damage as measured by the Comet assay, though the authors showed that 3-NBA could induce ROS production [16]. The authors suggested that the pro-oxidative properties of 3-NBA are related to the involvement of CYP1A1 and CYP1A2 enzymes in its metabolism. In our study, we used HPLC-MS/MS to measure specifically 8-oxodG, so our results cannot be compared directly with studies that used the Comet assay for analyses of oxidative DNA damage. We did not identify a significant increase in 8-oxodG levels, though there was some indication of possible 8-oxodG induction after 4-h treatment with 1-NP and 3-NBA. The lack of significant induction of oxidative DNA damage in our study may be explained (at least in part) by the fact that A549 cells exhibit greater resistance to this type of damage due to the a higher content of ferritin [32].

Our results further confirm the ability of 1-NP to induce ROS generation because the compound induced protein oxidation and, to a lesser extent, lipid peroxidation. There have been reports on the induction of lipid peroxidation by 1-NP in various experimental systems, including rat hepatocytes, mouse hepatoma cells and methyl linoleate [33,34,35]. However, our study is the first demonstrating the ability of 1-NP to induce oxidative damage to macromolecules other than DNA in A549 cells. In our study, 1-NP had the strongest pro-oxidant properties. Contrary to 1-NP, we did not observe any effect of 3-NBA exposure upon protein oxidation. Nevertheless, 3-NBA increased levels of lipid peroxidation, particularly after 24-h treatment when 15-F2t-IsoP levels were significantly higher than those in controls. This is the first report focusing on oxidative damage to lipids and proteins induced by 3-NBA in in vitro systems.

Finally, we analyzed mRNA expression of *COX2*, whose protein product catalyzes the synthesis of prostaglandins using arachidonic acid (AA) as a substrate. COX-2 is overexpressed during inflammation, and an association with increased oxidative stress and activation of nuclear factor-kappa B has been proposed [36]. In our study, 1-NP was a strong inducer of *COX2* expression, further confirming the pro-oxidant activity of this compound. However, there may be another role of COX2 that may affect detection of oxidative stress. 15-F2t-IsoP is formed from AA independent of COX2 [37]. We speculate that elevated expression of *COX2* could limit the cellular content of AA that would otherwise be available for 15-F2t-IsoP. This phenomenon would result in lower production of 15-F2t-IsoP after ROS attack. We observed a similar phenomenon in our previous study [29], but we did not have gene expression data to confirm our hypothesis. The present study showed significant down-regulation of *COX2* expression after 4-h treatment with B[a]P and 3-NBA, whereas 24-h treatment with 1-NP elicited very strong up-regulation of expression of this gene. It is interesting to note that twenty four-hour treatment with 1-NP had no effect on 15-F2t-IsoP levels, whereas a significant increase in 15-F2t-IsoP levels after 24-h incubation of cells with 3-NBA was not accompanied by up-regulation of *COX2* expression. This observation suggests that our hypothesis may be correct, but more experiments are needed to confirm it.

Finally, it should be noted that our results represent a typical in vitro study. Such studies are important as they help to investigate the mechanisms of action of various compounds in model systems. However, it is difficult to extrapolate the results to real-life conditions and estimate possible negative health effects in humans. First, in these studies cell lines that lack three-dimensional interactions of cells in tissues/organs of multicellular organisms are used. Second, the concentrations of the tested compounds in the ambient air are orders of magnitude lower than those used for in vitro tests (for B[a]P, the ambient concentrations are usually in the range of 0.1–10 ng/m^3^ which corresponds to femtomolar concentrations of the compound in the culture media; the ambient concentrations of 1-NP and 3-NBA are only in the tens of pg/m^3^ range). Moreover, these compounds are present in complex mixtures with many other chemicals which affects the interaction with the organism. Thus, our data should be regarded as a valuable contribution to understanding the mechanisms of action of PAHs and nitro-PAHs in a model of alveolar basal epithelial cells that specifically highlighted the role of oxidative damage to macromolecules. This process, along with bulky DNA adduct formation, may contribute to increased cancer risk in humans.

## 4. Materials and Methods

### 4.1. Test Compounds

B[a]P, 1-NP and 3-NBA were obtained from Sigma–Aldrich (Saint Louis, MO, USA). These compounds were dissolved in dimethyl sulfoxide (DMSO) and stock solutions were stored at −80 °C. For treatment, non-cytotoxic doses of each compound were used: B[a]P, 0.1 and 1 μM; 1-NP, 1 and 10 μM; 3-NBA, 0.5 and 5 μM.

### 4.2. Cell Cultures and Treatment

A549 cells (human adenocarcinoma alveolar basal epithelial cells, type II) were grown in Dulbecco’s Modified Eagle’s medium (DMEM) supplemented with 1.0 g/L glucose and pyruvate, 10% fetal bovine serum (FBS), 200 mM glutamine and gentamicin sulfate (10 mg/mL). Cells were cultivated in plastic cell-culture dishes (21 cm^2^) at 37 °C in an atmosphere of 5% CO_2_. After reaching 70%–80% confluence, the medium was replaced with fresh medium supplemented with 1% FBS. Test compounds were diluted with DMSO and added to the medium at test concentrations. Cells were treated for 4 h or 24 h. Each concentration was tested in triplicate in two independent experiments including control cell cultures incubated with DMSO only. The final concentration of DMSO was ≤0.1% of the total incubation volume. Harvested cells were washed thrice in phosphate-buffered saline (PBS) and stored at −80 °C until further processing. Cytotoxicity of test concentrations of B[a]P, 1-NP and 3-NBA in A549 cells was analyzed using a Lactate Dehydrogenase Cytotoxicity Assay kit (Bio Vision, Milpitas, CA, USA) according to manufacturer recommendations. Significant cytotoxicity was not observed at any of the test concentrations (Table 1).

### 4.3. DNA Isolation and DNA Adduct Analysis

DNA was isolated via phenol/chloroform/isoamylalcohol extraction and ethanol precipitation [38], and samples were stored at −80 °C until analysis. DNA adduct analysis was performed using ^32^P-postlabeling as described previously [39,40]. Briefly, DNA samples (6 μg) were digested with a mixture of micrococcal endonuclease (Sigma-Aldrich) and spleen phosphodiesterase (MP Biomedicals, Strasbourg, France) for 4 h at 37 °C. Nuclease P1 (Yamasa Corporation, Chiba-ken, Japan) was used for adduct enrichment. Labeled DNA adducts were resolved via multidirectional thin-layer chromatography (TLC) on 10 cm × 10 cm polyethylenimine–cellulose plates. The following solvent systems were used for TLC: D-1: 1 M sodium phosphate, pH 6.8; D-2: 3.8 M lithium formate, 8.5 M urea, pH 3.5; D-3: 0.8 M lithium chloride, 0.5 M Tris, 8.5 M urea, pH 8.0. Autoradiography was carried out at −80 °C for 24 h. Radioactivity of distinct adduct spots and diagonal radioactive zones was measured using liquid scintillation counting. To determine the exact amount of DNA in each sample, aliquots of the DNA enzymatic digest (1 μg of DNA hydrolyzate) were analyzed for nucleotide content using reverse-phase high performance liquid chromatography with UV detection, which simultaneously controlled for DNA purity. DNA adduct levels were expressed as relative DNA adduct levels per 10^8^ nucleotides. A BPDE-DNA adduct standard was run in duplicate in each postlabeling experiment to control for interassay variability.

### 4.4. RNA Isolation and Quality Control

Total RNA from lysed A549 cells was obtained using NucleoSpin RNA II (Macherey-Nagel GmbH & Co. KG, Düren, Germany) according to manufacturer instructions. RNA concentrations were quantified with a Nanodrop ND-1000 Spectrophotometer (Thermo Fisher Scientific, Waltham, MA, USA). RNA integrity was assessed using an Agilent 2100 Bioanalyzer (Agilent Technologies, Santa Clara, CA, USA). All samples had an RNA Integrity Number >9. Isolated RNA was stored at −80 °C until processing.

### 4.5. Analyses of mRNA Expression

Quantitative real-time polymerase chain reaction (qRT-PCR) was performed using a QuantiTect Probe RT-PCR kit (Qiagen, Hilden, Germany) on a 7900HT Fast Real-Time PCR System (Applied Biosystems, Waltham, MA, USA). Each RNA sample (1 μL, 300 ng) was mixed with 1 μL of primers (stock solution, 5 μM; Generi Biotech, Hradec Kralove, Czech Republic), 0.1 μL of fluorescence probe (stock solution: for NQO1, POR, AKR1C2, COX2: 10 μM, Roche, Mannheim, Germany; for CYP1A1 and CYP1B1: 1 μM, Generi Biotech), 0.1 μL RT mix, and 5 μL RT-PCR mix and incubated 30 min at 55 °C. qRT-PCR included a 15-min incubation at 95 °C and 40 cycles of 15-sencond incubation at 95 °C followed by 1-min incubation at 60 °C. Primer sequences are shown in Table 2.

### 4.6. Analyses of 8-oxo-7,8-Dihydro-2′-deoxyguanosine (8-oxodG)

DNA was isolated from cell pellets using a DNeasy Blood and Tissue kit (Qiagen, Hilden, Germany), denaturated by heating, and cooled rapidly on ice. Denatured samples were incubated with 4 μL of Nuclease P1 (1 U/1 μL; Sigma–Aldrich) for 1 h at 37 °C and then with 2 μL of alkaline phosphatase (3.5 mg/30 μL; Sigma–Aldrich) for 1 h at 37 °C. The reaction was terminated by addition of 8 μL of 3 M sodium acetate (pH 5). Samples were stored at −80 °C. The ratio of 8-oxodG/2’-deoxyguanosine (dG) was determined using liquid chromatography-tandem mass spectrometry (LC/MS-MS). A 1200 chromatographic system (Agilent Technologies) comprised a binary pump, vacuum degasser, autosampler, thermostatted column compartment, and a UV detector. Separation of analytes was carried out using a ZORBAX Eclipse Plus C18, 2.1 × 150 mm, 3.5 μm particle-size column (Agilent Technologies) with a 15-min linear gradient from 2.5% to 23% of methanol. The flow rate of the mobile phase was 0.2 mL/min, and the column temperature was set at 45 °C. A triple quadrupole mass spectrometer (6410 Triple Quad LC/MSl Agilent Technologies) with an electrospray interface (ESI) was used for detection of 8-oxodG. The mass spectrometer was operated in positive ion mode. Multiple reaction monitoring (MRM) with the mass transition *m*/*z* 284.1 → *m*/*z* 168.1 was used for quantification. dG was detected with a UV detector at 260 nm.

### 4.7. Preparation of Cell Lysates; Analyses of Lipid Peroxidation and Protein Oxidation

Analyses of lipid peroxidation and protein oxidation were carried out using enzyme-linked immunoassays (ELISAs) as described previously [29] with modification. Cells stored at −80 °C were thawed and mixed with 100 μL of CelLytic Reagent (Sigma–Aldrich) and mixed vigorously for 15 min at room temperature on a shaker. Lysates were centrifuged at 18,000× *g* for 15 min at 4 °C. Supernatants were transferred to a new tube and stored at −80 °C or used directly for analyses of total protein concentration using a Bicinchoninic Acid kit (Sigma–Aldrich). The concentration of 15-F_2t_-isoprostane (15-F2t-IsoP) in cell lysates was analyzed by use of immunoassay kits from Cayman Chemical Company (Ann Arbor, MI, USA). First, membrane-bound 15-F2t-IsoP was hydrolyzed and samples purified. Cell lysates containing 100 μg of protein were diluted with dH_2_O to 100 μL. Then, 100 μL of 15% KOH was added and the samples vortex-mixed and incubated for 60 min at 40 °C. pH of the samples was adjusted by addition of 300 μL of 1 M KH_2_PO_4_; 100 μL of column buffer (containing 13.6 g KH_2_PO_4_, 29.2 g NaCl, 0.5 g NaN_3_ per 1000 mL, pH 7.4) was added, and samples were mixed. The next step included addition of 50 μL of Isoprostane Affinity Sorbent (Cayman Chemical Company) and incubation for 60 min at room temperature on a shaker. After incubation, samples were centrifuged for 1 min at 5000× *g* and supernatants removed by pipetting. 15-F_2t_-isoprostane bound to sorbent was washed with 1 mL of dH_2_O and eluted from the sorbent by re-suspension in 0.5 mL of elution solution (95% ethanol). Then, samples were stored in elution solution at −80 °C until analyses. Before the assay, samples were vacuum-dried, re-suspended in 110 μL of enzyme immunoassay (EIA) buffer (supplied with the 15-F_2t_-IsoP kit) and used immediately for ELISA, which was performed according to manufacturer instructions.

Protein oxidation was analyzed using a published protocol [29]. Briefly, cell lysates were diluted with PBS to a final protein concentration of 2 mg/mL. Ten microliters of samples were mixed with 10 μL of 2,4-dinitrophenylhydrazine (DNPH) solution (derivatization solution). Then, samples were incubated at room temperature in the dark for 45 min and vortex-mixed every 10–15 min. Derivatization was stopped by addition of 30 μL of 2 M Tris, and samples were mixed on a shaker and centrifuged briefly. Of the derivatized samples, 6.25 μL were added to 1.25 mL of coating buffer, mixed and used for coating ELISA plates. The ELISA was performed as described in [29].

To account for the biological variability of markers of oxidative stress within triplicates, results were normalized to the basal level of oxidative damage in the negative control sample, and these relative numbers were used for statistical analyses. In addition, data in the figures are shown as relative values of oxidative damage.

### 4.8. Statistical Analyses

Raw data from qRT-PCR were analyzed using SDS Relative Quantification v2.3 (Applied Biosystems). Expression levels of analyzed genes were normalized to those of the reference gene porphobilinogendeaminase (PBDG; obtained from a kit from Generi Biotech). mRNA expression was compared to that in control samples treated with DMSO only. Changes in relative gene expression were calculated using the 2^−ΔΔ*C*t^ method [41].

For statistical analyses of data on DNA adducts, oxidative stress, and mRNA expression, SPSS v20.0 (IBM, Armonk, NY, USA) was used. Data followed a normal distribution, so the Student’s *t*-test was used for comparisons between groups. Figures showed the mean value of DNA adducts, relative value of markers of oxidative damage, and the mean fold-change ± standard deviation of mRNA expression.

## 5. Conclusions

Our data demonstrated the very strong genotoxic effects of 3-NBA in A549 cells even after short incubation; the effect of 1-NP was limited. All test compounds seemed to induce ROS generation, though oxidative damage to macromolecules was inconsistent. The most pronounced effects were observed for protein oxidation induced by 1-NP and for 15-F2t-IsoP induced by 3-NBA. In our experimental system, nitro-PAHs (particularly 1-NP) were stronger oxidants than B[a]P.

## Figures and Tables

**Table 1:** Cytotoxicity of test compounds after treatment of A549 cells with selected concentrations of B[a]P, 1-NP and 3-NBA for 4 h or 24 h.

Test Compound	Concentration (μM)	Cytotoxicity (%)	
		4 h	24 h
B[a]P	0.1	ND	ND
	1.0	ND	ND
1-NP	1.0	ND	ND
	10	ND	0.6
3-NBA	0.5	ND	ND
	5.0	ND	2.0

ND, not detectable.

**Table 2:** Analyzed genes and primer sequences used in qRT-PCR.

Gene	RefSeq ID		Primer Sequence
CYP1A1	NM_000499	Forward	CACCATCCCCCACAGCAC
		Reverse	TTACAAAGACACAACGCCCC
CYP1B1	NM_000104	Forward	GCTTTTTCTCTTCATCTCCATC
		Reverse	TTCATTTTCGCAGGCTCATTTG
ALDH3A1	NM_001135168	Forward	TCTTGGCTCTTGCCGTTC
		Reverse	CGCTGATCTTGCTCATGG
COX2	NM_000963	Forward	CTTCACGCATCAGTTTTTCAAG
		Reverse	TCACCGTAAATATGATTTAAGTCCAC
AKR1C2	NM_001135241	Forward	AGCACCTCCACCTTCTCTCTC
		Reverse	AGGTGAAGGGGAAGTAAGCAT
NQO1	NM_000903	Forward	ATGTATGACAAAGGACCCTTCC
		Reverse	TCCCTTGCAGAGAGTACATGG
POR	NM_000941	Forward	GAGACCCCACCGACAATG
		Reverse	TGGCATTGAAGTGCTCGTAG

## Abbreviations

AA	Arachidonic Acid
AKR1C2	Aldo-Keto Reductase
B[a]P	Benzo[a]pyrene
COX2	Cyclooxygenase-2
ER stress	Endoplasmic Reticulum Stress
15-F2t-IsoP	15-F_2t_-Isoprostane
3-NBA	3-nitrobenzanthrone
N-OH-ABA	*N*-hydroxy-3-aminobenzanthrone
1-NP	1-nitropyrene
NQO1	NAD(P)H:QuinoneOxidoreductase
8-oxodG	8-oxo-7,8-dihydro-2′-deoxyguanosine
PAHs	Polycyclic Aromatic Hydrocarbons
POR	NAPDH: Cytochrome P450 Oxidoreductase
ROS	Reactive Oxygen Species
XO	Xanthine Oxidase
